# Exome sequence analysis of Kaposiform hemangioendothelioma: identification of putative driver mutations*

**DOI:** 10.1590/abd1806-4841.20165026

**Published:** 2016

**Authors:** Sho Egashira, Masatoshi Jinnin, Miho Harada, Shinichi Masuguchi, Satoshi Fukushima, Hironobu Ihn

**Affiliations:** 1 Faculty of Life Sciences, Kumamoto University – Kumamoto, Japan

**Keywords:** Exome, Hemangioendothelioma, Mutation

## Abstract

**BACKGROUND:**

Kaposiform hemangioendothelioma is a rare, intermediate, malignant tumor. The tumor's etiology remains unknown and there are no specific treatments.

**OBJECTIVE:**

In this study, we performed exome sequencing using DNA from a Kaposiform hemangioendothelioma patient, and found putative candidates for the responsible mutations.

**METHOD:**

The genomic DNA for exome sequencing was obtained from the tumor tissue and matched normal tissue from the same individual. Exome sequencing was performed on HiSeq2000 sequencer platform.

**RESULTS:**

Among oncogenes, germline missense single nucleotide variants were observed in the TP53 and APC genes in both the tumor and normal tissue. As tumor-specific somatic mutations, we identified 81 candidate genes, including 4 nonsense changes, 68 missense changes and 9 insertions/deletions. The mutations in ITGB2, IL-32 and DIDO1 were included in them.

**CONCLUSION:**

This is a pilot study, and future analysis with more patients is needed to clarify: the detailed pathogenesis of this tumor, the novel diagnostic methods by detecting specific mutations, and the new therapeutic strategies targeting the mutation.

## INTRODUCTION

According to the International Society for the Study of Vascular Anomalies (ISSVA) classification, vascular anomalies are classified into vascular tumors with proliferative changes in endothelial cells and vascular malformation characterized by abnormal dilation of blood vessels without proliferative change. The former includes infantile hemangioma, tufted angioma and Kaposiform hemangioendothelioma (KHE).

The disease concept of KHE was first proposed by Zukerberg in 1993.^1^ The intermediate malignant tumor is a rare, locally aggressive, vascular tumor that usually occurs during childhood. KHE may be associated with Kasabach-Merritt syndrome (KMS), a severe condition characterized by profound thrombocytopenia and hemorrhage. Because its mortality has been reported as 24%, it is necessary to clarify this tumor's detailed pathogenesis to develop early diagnostic methods and new therapeutic strategies. ^2^

The tumor's etiology remains unknown. Diagnosing KHE depends on histopathologic examination. The tumor is filled with slit-like vascular spaces and intracellular lumina, sometimes infiltrating muscles and subcutaneous fat tissue. The neoplastic tumor cells at the periphery of the tumor were positive for CD31, CD34 and lymphatic markers (D2-40, Prox1 or LYVE-1). Nevertheless, these cells were negative for GLUT-1 (infantile hemangioma marker) and HHV-8 (Kaposi's sarcoma marker).

No treatment guidelines exist for KHE. Previous reports have indicated that complete surgical excision is the most reliable treatment.^3^ When surgical excision is impossible, combined therapy, including high-dose steroids, cytotoxic agent (vincristine or interferon-α) and cyclophosphamide, is required. Furthermore, a small number of studies have reported the effects of sirolimus, β-blocker, radiotherapy and embolization.^4,5^ However, the tumor is basically resistant to such treatments and complete remission is usually difficult to achieve.

Recently, advances in sequencing methods have enabled the identification of driver genes in many cancers. For example, mutations in BRAF are found in malignant melanoma, and BRAF inhibitors have already been utilized as novel treatments. Nonetheless, no studies exist on the causative genes in KHE. In this study, we performed exome sequencing of KHE and found putative candidates for the responsible mutations.

## METHOD

### Case

A 2-year-old Japanese boy visited our hospital to treat eruptions. His parents had noticed erythemas on his lower abdominal area at birth. His eruption was monitored at another hospital but the lesion gradually became larger, violaceous and painful.

**Figure 1:**
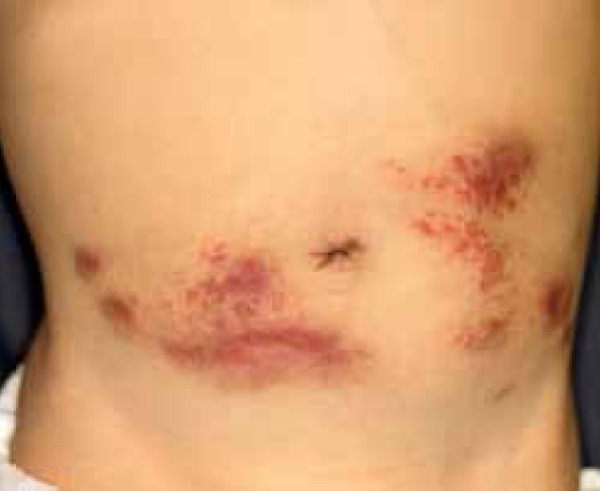
Violaceous and indurated multiple lesions in the abdomen

**Figure 2:**
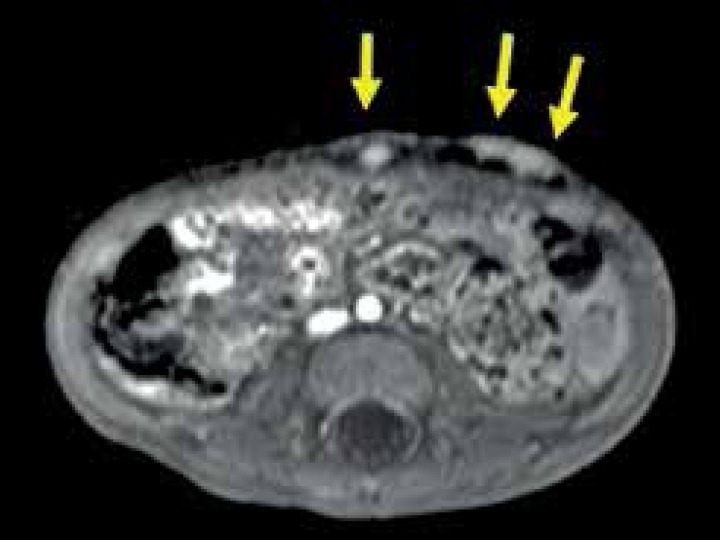
MRI of the abdomen. Arrows indicate multiple, subcutaneous nodules enhanced by intravenous contrast agent

**Figure 3:**
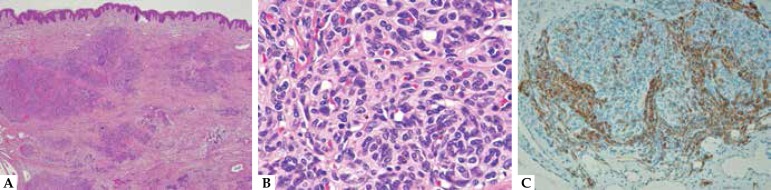
**A.** Hematoxylin-eosin staining of biopsy specimen from cutaneous lesion. Multiple nests of tumor cells in the dermis and fat tissue. Magnification x40; **B.** Hematoxylin-eosin staining of biopsy specimen from cutaneous lesion. The tumor cells were spindle-shaped or round, containing interspersed capillaries with slit-like lumens. There was slight nuclear variation. Magnification x400; **C.** D2-40 staining showing positiveness in peripheral area of tumor nests, but not in the surrounding dilated vessels. Magnification x200

On physical examination at the first visit, multiple, indurated nodules and erythemas merged to become geographical (Figure 1). His general condition was good and there was no record of any similar condition in his family history. Laboratory data including platelet count, prothrombin time, activated partial thromboplastin time and international normalized ratio were within the normal range. T2-weighted magnetic resonance imaging (MRI) revealed multiple subcutaneous nodules, enhanced by intravenous contrast agent (Figure 2). No apparent invasion into the muscle or fascia was noted. Hematoxylin and Eosin (H&E)-stained sections of skin biopsy specimen from the lesion showed multiple nests of tumor cells in the dermis and fat tissue without epidermal change (Figure 3). The tumor cells were spindle-shaped or round, containing interspersed capillaries with slit-like lumens and red blood cells. Slight nuclear variation was observed, but no significant nuclear mitosis, atypia or necrosis. Histopathologically, these tumor cells were positive for CD31 and CD34, but negative for GLUT-1. In addition, D2-40 was positive in the peripheral area of tumor nests and negative in the surrounding dilated vessels (Figure 3).

Based on the above findings, a diagnosis of KHE was made. Since the tumor was limited to the lower abdomen without muscle infiltration or distant metastasis, and because the patient was not accompanied with KMS, the decision was taken to treat the tumor by serial excision. Three times resection removes almost all the lesions and the pain disappears.

### DNA purification

The genomic DNA for exome sequencing was obtained from the tumor tissue and matched normal tissue from the same individual, using the DNA mini kit (Qiagen, Valencia, CA). Institutional review board approval and informed consent were obtained, conforming to the Declaration of Helsinki.

### Library preparation and sequencing

Exome sequencing was performed in accordance with the protocol provided by InfoBio (Tokyo, Japan). DNA was treated with the TruSeq DNA Sample Prep kit and TruSeq Exome Enrichment kit (Illumina, San Diego, CA) to provide libraries, which were sequenced on the HiSeq2000 sequencer platform (Illumina) in a paired-end 100bp configuration. Image analysis and base calling were performed using the Illumina pipeline.

The clean and trimmed reads were aligned to the reference human genome (UCSC hg19) using Burrows-Wheeler Aligner (BWA) on default settings. The bioinformatic analysis for detecting single nucleotide variants (SNVs) and inserts/deletions was performed using the Samtools (v1.0) software program and annotated according to dbSNP.

## RESULT

High-quality DNAs were isolated from excised tumor tissue and matched normal tissue was derived from the same individual; these were then analyzed using paired-end exome sequences on the Illumina HiSeq2000 platform.

When compared with the reference human genome (UCSC hg19), there were 80,062 nucleotide changes in the normal tissue, and 73,878 changes in the tumor tissue. Among them, 68,342 changes were common to both tissues. We tried to extract SNVs and inserts/deletions likely to be associated with the pathogenesis.

First, we focused on oncogenes. Among APC, BCL2, TP16, FOS, MYC, TP53, RAS and VHL, as shown in table 1, the missense SNVs with a T-to-A transition or a G-to-C transition were observed in the TP53 or APC genes of tumor tissue, respectively. However, these SNVs were not tumor-specific changes because they were also noted in the matched normal tissue. The APC sequence change rs459552 is present in 86% of the population, according to allele frequency from the 1,000 genomes project of October 2011, indicating that SNV represents a common variant. However, although TP53 T-to-A transition rs1042522 is reportedly found in 60%, it was reported in several malignant tumors, and germline mutations in rs1042522 are thought to be associated with Li–Fraumeni syndrome, which is characterized by a hereditary predisposition to several cancers.^6,7^ Accordingly, this germline SNV may be the pathologic change.

**Table 1:** Changes observed in oncogenes

Gene	Chr	Chr_start	Region	Homo/Hetero	Ref	Alt	Amino acid change	Genetic status
APC	5	112176756	exonic	Homo	T	A	V→D	germline
TP53	17	25358943	exonic	Homo	G	C	R→P	germline

Chr; chromosome, Chr_start: chromosome start site, Homo/Hetero; heterozygosity status, Ref; reference allele, Alt; alternative allele.

Subsequently, the team tried to uncover tumor-specific somatic mutations. Among the nonsynonymous SNVs, stop/gain SNVs or insertions/deletions observed only in tumor tissue and not in normal tissue, 81 candidate genes were selected by the following criteria: 1) frequency of under 1%; and 2) alternative depth/reference depth ratio of above 0.5 in heterozygous changes (Tables 2-4). Missense changes were seen in 68 genes (Table 2). In addition, PTRF, OLFML2A, WDR81, or DIDO1 were detected as stop/gain SNVs (Table 3). Eight out of the 9 insertions/deletions did not cause frameshift and only G insertion in the IL-32 gene resulted in frameshift (Table 4).

**Table 2:** Missense somatic changes

Gene	Chr	Chr_start	Ref	Alt	Homo/Hetero	Ref_depth	Alt_depth
AQP11	chr11	77301121	G	A	Homo	0	2
CHST6	chr16	75513276	G	T	Hetero	3	2
DHPS	chr19	12792439	C	A	Hetero	3	2
GAL3ST2	chr02	242742895	C	A	Hetero	3	2
PTPN21	chr14	88945674	G	T	Hetero	3	2
PYDC1	chr16	31228226	C	A	Hetero	3	2
ZBTB4	chr17	7366209	A	G	Hetero	3	2
SELRC1	chr01	53158524	A	C	Hetero	10	8
FAM135A	chr06	71187020	A	C	Hetero	11	9
CEMP1	chr16	2580996	T	G	Hetero	4	4
GLB1L	chr02	220107628	C	A	Hetero	2	2
NFIC	chr19	3435089	G	T	Hetero	2	2
R3HDM4	chr19	899473	C	G	Hetero	2	2
RASSF1	chr03	50375431	T	G	Hetero	5	7
PLEKHH3	chr17	40824327	G	T	Hetero	1	2
ZNF512B	chr20	62594752	C	A	Hetero	1	2
GPRC5B	chr16	19883282	G	A	Hetero	1	3
ITPRIPL2	chr16	19126384	C	A	Homo	0	2
ITPRIPL2	chr16	19126388	C	A	Homo	0	2
LRRC24	chr08	145749537	C	A	Homo	0	2
MBD3L5	chr19	7032880	A	G	Homo	0	2
PTPMT1	chr11	47587479	C	A	Homo	0	2
TFR2	chr07	100228635	T	C	Homo	0	2
TTLL4	chr02	219603798	A	C	Homo	0	3
ZAR1L	chr13	32885737	G	T	Homo	0	2
ABCB11	chr02	169828367	T	G	Hetero	15	8
RETSAT	chr02	85571228	G	C	Hetero	13	7
CLIP1	chr12	122812693	G	T	Hetero	7	4
FAM75A6	chr09	43627675	C	A	Hetero	44	26
DNAH12	chr03	57438710	C	A	Hetero	13	8
CRLF1	chr19	18705064	C	T	Hetero	3	2
GPIHBP1	chr08	144297240	C	A	Hetero	3	2
GRID2IP	chr07	6542766	C	A	Hetero	3	2
ITGB2	chr21	46309368	C	A	Hetero	3	2
PABPC1	chr08	101719004	G	A	Hetero	35	24
OR11H1	chr22	16449784	C	A	Hetero	31	23
CATSPERG	chr19	38851455	A	C	Hetero	4	3
CLEC18B	chr16	74451970	G	C	Hetero	4	3
PABPC1	chr08	101719201	A	G	Hetero	25	20
RASAL1	chr12	113544922	A	C	Hetero	5	4
CDC27	chr17	45234417	A	G	Hetero	41	34
SPATA20	chr17	48626182	A	C	Hetero	6	5
MUC7	chr04	71347171	C	T	Hetero	20	17
BCOR	chrX	39931672	C	A	Hetero	2	2
C19orf57	chr19	14001212	C	A	Hetero	2	2
CARD9	chr09	139264769	G	T	Hetero	2	2
CRB1	chr01	197313422	G	A	Hetero	3	3
DDX18	chr02	118572361	A	C	Hetero	3	3
IL22RA1	chr01	24469556	G	T	Hetero	2	2
MAD1L1	chr07	2108930	G	T	Hetero	2	2
MRGPRE	chr11	3249491	A	C	Hetero	2	2
OBSCN	chr01	228400288	G	T	Hetero	2	2
SNED1	chr02	241974126	G	T	Hetero	2	2
ARSH	chrX	2936675	T	G	Hetero	5	8
CACNA1I	chr22	40045803	G	T	Hetero	1	2
LRFN4	chr11	66627620	G	T	Hetero	1	2
MRC2	chr17	60767030	C	A	Hetero	1	2
SHROOM2	chrX	9862832	G	T	Hetero	1	2
LAG3	chr12	6884651	A	C	Hetero	3	7
OBSL1	chr02	220422281	C	T	Hetero	1	3
TTN	chr02	179419226	A	C	Hetero	1	3
USP49	chr06	41774685	C	G	Hetero	1	3
GPRIN2	chr10	46999604	A	G	Hetero	2	12
ADAMTS7	chr15	79058378	A	G	Homo	1	8
FAM83E	chr19	49116421	G	T	Homo	0	2
LILRB3	chr19	54725745	A	G	Homo	0	4
NKX6-2	chr10	134598908	C	A	Homo	0	2
PFKL	chr21	45744745	G	T	Homo	0	2

Chr; chromosome, Chr_start: chromosome start site, Ref; reference allele, Alt; alternative allele, Homo/Hetero; heterozygosity status, Ref_depth; read depth of reference allele, Alt_depth; read depth of alternative allele.

**Table 3:** Nonsense somatic changes

Gene	Chr	Chr_start	Ref	Alt	Homo/Hetero	Ref_depth	Alt_depth
PTRF	chr17	40557025	C	A	Hetero	3	2
OLFML2A	chr09	127549304	C	A	Hetero	2	2
DIDO1	chr20	61542820	C	A	Homo	0	2
WDR81	chr17	1636925	G	T	Homo	0	2

Chr; chromosome, Chr_start: chromosome start site, Ref; reference allele, Alt; alternative allele, Homo/Hetero; heterozygosity status, Ref_depth; read depth of reference allele, Alt_depth; read depth of alternative allele.

**Table 4:** Insertion/deletion somatic changes

Gene	Chr	Chr_start	Ref	Alt	Homo/Hetero	Ref_depth	Alt_depth
IL32	chr16	3119303	-	G	Hetero	25	15
FAM48B1	chrX	24382426	GCT	-	Homo	0	2
ATXN1	chr06	16327915	ATG	-	Hetero	1	3
HAVCR1	chr05	156479571	CATTGGAACAGTCGT	-	Homo	0	20
PCDHB10	chr05	140574177	GGCCGA	-	Homo	0	4
POLI	chr18	51795967	CGA	-	Homo	0	5
CCDC66	chr03	56650056	-	TCT	Homo	0	31
FAM83G	chr17	18874687	-	GGG	Homo	0	2
NR1H2	chr19	50881831	-	CAG	Homo	0	7

Chr; chromosome, Chr_start: chromosome start site, Ref; reference allele, Alt; alternative allele, Homo/Hetero; heterozygosity status, Ref_depth; read depth of reference allele, Alt_depth; read depth of alternative allele.

## DISCUSSION

KHE is a rare, vascular tumor experienced in childhood that invades skin and cutaneous tissues locally. The prognosis of KHE accompanied with KMS is rather poor. Specific driver mutations of the tumor remain unknown. This is the first study to investigate mutations using the exome sequence, and we described several putative causing mutations of KHE.

For instance, the putative germline mutation seen in both tumor tissue and matched normal tissue was TP53 rs1042522. Given that TP53-deficient mice developed several spontaneous tumors including angiosarcoma, another malignant vascular tumor, TP53 mutation may play a role in the tumorigenesis of vascular tumors.^8^ However, since this SNV is seen in about 60% of the population, there is a possibility that "second-hit" somatic mutation is also necessary for tumorigenesis. Another vascular anomaly, mucocutaneous venous malformation is caused by the combination of germline substitutions in the endothelial cell tyrosine kinase receptor TIE2 and the somatic 'second hit' lesion-restricted mutation of TIE2.^9^ Similarly, we previously indicated germline heterozygous SNV in KDR and TEM8 as the risk mutations for infantile hemangioma.^10^ As infantile hemangioma typically appears on the head or face around the second week of life, the hypothesis proposed is that the clonal expansion of endothelial cells within the lesions may be a consequence of somatic events such as microvessel trauma during delivery.

We also identified 81 genes as candidates for somatic mutation. For example, ITGB2 has been strongly implicated in angiogenesis, suggesting the possible association with the pathogenesis of KHE. IL-32, which possesses heterozygous frameshift in KHE tissue, has also been known as a angiogenesis-related cytokine.^11,12^ Furthermore, we found 4 nonsense mutations only in tumor tissue, including the DIDO1 gene, which is up-regulated by apoptotic signals and encodes a cytoplasmic protein that translocates to the nucleus upon apoptotic signal activation. DIDO1 is considered a tumor suppressor gene in myeloid cells, and thought to be significantly involved in the pathogenesis of myelodysplastic syndrome, a malignant blood disorder.^13^ Accordingly, the DIDO1 gene may also be involved in the tumorigenesis of KHE.

## CONCLUSION

These mutations have not been reported so far, which is consistent as driver mutations of the rare tumor. Potentially, multiple genetic abnormalities - rather than a single abnormality – may be involved in KHE. For instance, germline TP53 SNV and the somatic DIDO1 gene change may cooperate to induce tumorigenesis.

As limitations to this study, 'germline' mutation of TP53 may be due to the contamination of normal tissue and tumor tissue, because the parent DNA is not determined. In addition, the result of exome analysis was not confirmed by the sanger method. Since KHE is a rare tumor, the team was unable to collect other samples. This is a pilot study, and future analysis with more patients is needed to clarify: this tumor's detailed pathogenesis, the novel diagnostic methods by detecting specific mutations, and the new therapeutic strategies targeting the mutation.
